# Inside the family meal: a thematic analysis of session two in Maudsley family-based treatment for anorexia nervosa

**DOI:** 10.1186/2050-2974-2-S1-O2

**Published:** 2014-11-24

**Authors:** Kate Godfrey, Paul Rhodes, Jane Miskovic-Wheatley, Andrew Wallis, Simon Clarke, Michael Kohn, Stephen Touyz, Sloane Madden

**Affiliations:** 1 Clinical Psychology Unit, The University of Sydney, Sydney, Australia; 2 Eating Disorder Service, The Children's Hospital at Westmead, Sydney, Australia; 3 Adolescent Unit, Westmead Hospital, Sydney, Australia

## Aim

The family meal is a key session in Maudsley family-based treatment for anorexia nervosa. This study aimed to determine whether there are different types of family meal and whether within session processes (i.e., strategies used by the therapist and each family member) differ according to meal type.

## Method

Thirty video-recorded family meal sessions from a randomised controlled trial were transcribed and analysed using thematic analysis. Transcripts were initially coded for units of meaning. Themes were then extracted and analysed to create a greater understanding of the processes occurring during the session.

## Results

Two types of family meal were identified. The first was characterised by processes that were consistent with the Maudsley model, and resulted in the patient eating one mouthful more than they were prepared to. The second was defined by processes that were mixed in terms of their consistency with the model, and resulted in the patient eating what was asked of them with little to no difficulty. Therapist and family avoidance differentiated the second meal type from the first.

## Discussion

Avoidance seemed to diminish the therapeutic impact of the family meal for a significant number of families. Strategies to challenge avoidance during the session are suggested.

This abstract was presented in the **Peter Beumont Young Investigator award finalist** stream of the 2014 ANZAED Conference.

